# An allosteric link connecting the lipid-protein interface to the gating of the nicotinic acetylcholine receptor

**DOI:** 10.1038/s41598-018-22150-x

**Published:** 2018-03-01

**Authors:** Jaimee A. Domville, John E. Baenziger

**Affiliations:** Department of Biochemistry, Microbiology, and Immunology, University of Ottawa, Ottawa, ON K1H 8M5 Canada

## Abstract

The mechanisms underlying lipid-sensing by membrane proteins is of considerable biological importance. A unifying mechanistic question is how a change in structure at the lipid-protein interface is translated through the transmembrane domain to influence structures critical to protein function. Gating of the nicotinic acetylcholine receptor (nAChR) is sensitive to its lipid environment. To understand how changes at the lipid-protein interface influence gating, we examined how a mutation at position 418 on the lipid-facing surface of the outer most M4 transmembrane α-helix alters the energetic couplings between M4 and the remainder of the transmembrane domain. Human muscle nAChR is sensitive to mutations at position 418, with the Cys-to-Trp mutation resulting in a 16-fold potentiation in function that leads to a congenital myasthenic syndrome. Energetic coupling between M4 and the Cys-loop, a key structure implicated in gating, do not change with C418W. Instead, Trp418 and an adjacent residue couple energetically with residues on the M1 transmembrane α-helix, leading to a reorientation of M1 that stabilizes the open state. We thus identify an allosteric link connecting the lipid-protein interface of the nAChR to altered channel function.

## Introduction

With increasing numbers of available structures, renewed interest has focused on the mechanisms underlying the functional sensitivities of membrane proteins to lipids. In some cases, the modes of lipid action are relatively clear in that binding sites in the transmembrane domain (TMD) have been identified, with the bound lipids acting as classic allosteric modulators. In other cases, the entire lipid-facing surface of a TMD may act as an allosteric “site” that is sensitive to the physico-chemical properties of the lipid bilayer, with changes in bilayer physico-chemical properties translated into altered function through complex mechanisms that likely involve altered TMD helix-helix packing^1^. In all cases, a central goal is to elucidate how changes at the lipid-protein interface are translated through the TMD to influence the structures that define protein function.

The functional sensitivity of the muscle-type nicotinic acetylcholine receptor (nAChR), a pentameric ligand-gated ion channel (pLGIC), to lipids has been known since the earliest attempts at isolating the receptor from the electric fish, *Torpedo*. To preserve function, the nAChR must be purified in the presence of exogenous lipids and reconstituted into membranes with a particular lipid composition^2–5^. Both cholesterol and anionic lipids promote nAChR function, although neither is absolutely required^6^. Cholesterol and anionic lipids influence function by stabilizing different proportions of activatable (resting) versus non-activatable (desensitized and uncoupled) conformations, as well as by controlling the transitions between these states^7,8^. The molecular details of how lipids interact with and thus stabilize one conformation or conformational transition state over another, however, remain poorly defined^9,10^.

**Figure 1:**
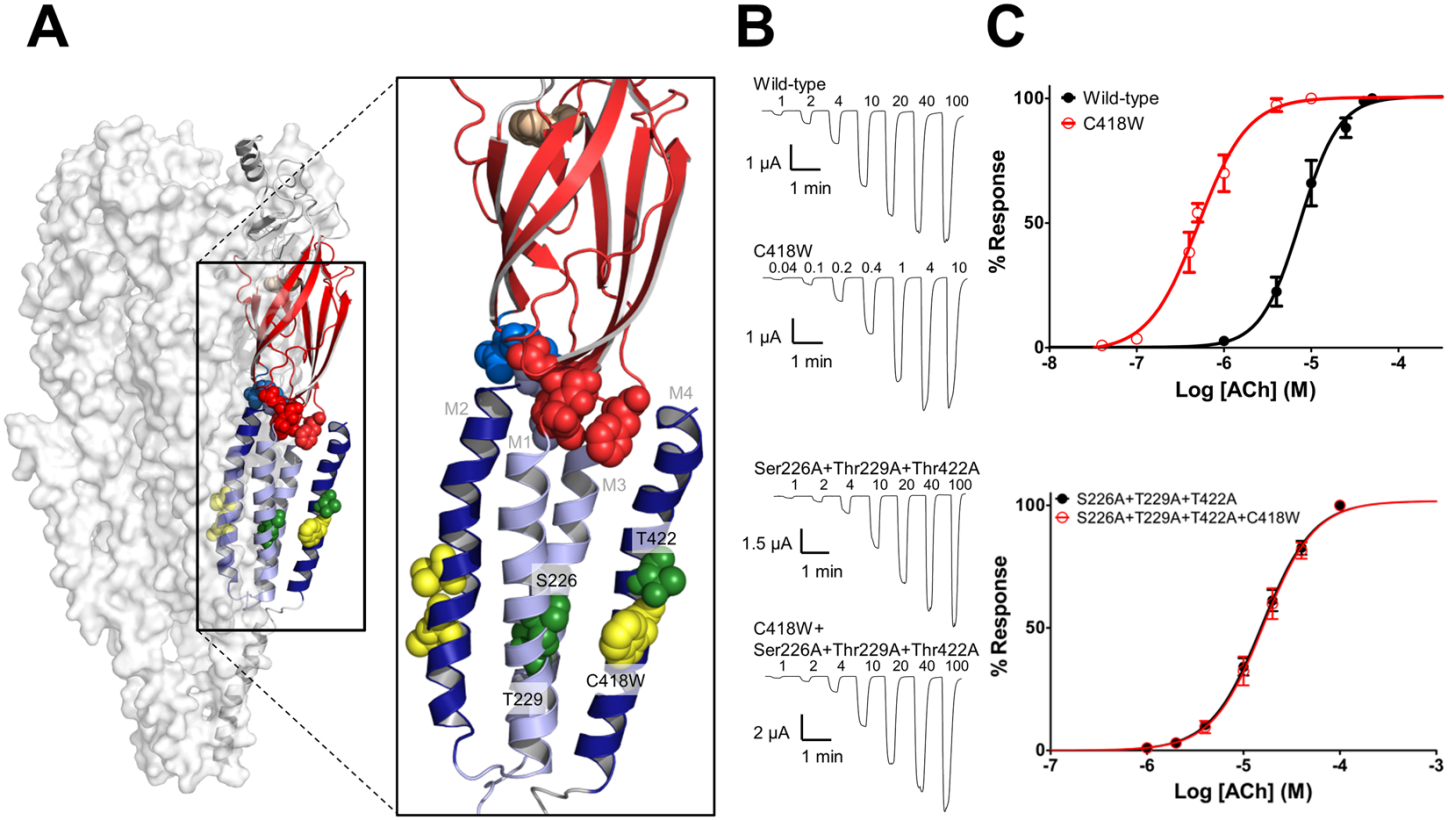
αM4 C418W potentiates WT-nAChR function. (**A**) Structure of the *Torpedo* nAChR (PDB: 2BG9) with a single α-subunit shown in a cartoon representation. The β strands in the extracellular domain are colored red, while the transmembrane α-helices are colored either dark (M2 and M4) or light (M1 and M3) blue. Residues critical for either channel function or C418W-induced potentiation are shown as spheres. Illustrated residues implicated in channel function include the binding site Trp149 (tan), the β1-β2 loop Glu45 and Val46 (sky blue), the Cys-loop Phe135 and Phe137 (red), the pre-M1 Arg209 and Leu210 (red), and the M2-M3 linker Pro272 and Leu273 (light blue). Other highlighted residues include Leu251 and Val255, which line the transmembrane pore and form the hydrophobic gate (yellow), and the lipid-facing C418W mutation (yellow). Ser226, Thr229 and Thr422 are implicated in C418W-induced potentiation (green). (**B**) Representative whole cell currents elicited using the noted concentrations of acetylcholine (μM) for WT-nAChR and mutant nAChRs. (**C**) Averaged dose response curves showing the potentiation of channel function in C418W-nAChR versus WT-nAChR (top). The triple Ala mutant (Ser226A + Thr229A + Thr422A) abolishes C418W-induced potentiation (bottom). Error bars represent standard deviation, n ≥ 8.

Considerable work has focused on the M4 α-helix in each subunit of the nAChR as the transducer of lipid-protein interactions^11^. M4 is the outermost α-helix in the TMD of each subunit and thus forms the primary contact with lipids (Fig. 1)^12^. Mutations along the lipid-facing surface of M4 highlight the functional sensitivity of the nAChR to altered M4-lipid interactions^13–19^. Lipids are also bound at the interface between M4 and adjacent TMD α-helices, M1 and M3, in the crystal structure of the prokaryotic pLGIC, GLIC^20,21^.

M4 likely influences channel function via its interactions with the adjacent α-helices, M1 and M3^14,15^. Supporting this hypothesis, amino acid substitutions that promote more extensive M4-M1/M3 interactions in the prokaryotic pLGIC, ELIC, lower agonist EC_50_ values, suggesting enhanced coupling between the agonist binding site and channel gate^22^. These substitutions at the M4-M1/M3 interface also reduce the functional sensitivity of ELIC to a potentiating amino acid mutation on the M4 lipid-facing surface, and render ELIC functionally insensitive to bilayers with different lipid compositions^22–24^. In addition, thicker bilayers, which likely enhance M4-M1/M3 interactions, promote conformational transitions from “uncoupled” to agonist-responsive conformations^7,25^.

How could altered M4-M1/M3 interactions ultimately influence channel gating? The most obvious link between M4 and those structures involved in channel gating occurs at the C-terminus, where M4 interacts with the β6-β7 loop or “Cys-loop”^26^, a key structure implicated in channel function^27,28^. A lipid-dependent change in M4 - Cys-loop interactions could lead to an altered Cys-loop conformation that promotes coupling between the agonist site and the channel gate^25^. In support of this model, increasing evidence suggests that M4 - Cys-loop interactions are important in both the folding and function of some pLGICs^29–32^. Although ELIC functions in their absence, enhanced M4 - β6-β7 loop interactions promote channel function^31^.

Here, we use a Cys-to-Trp mutation at position 418 in the human muscle nAChR to investigate how changes at the lipid-protein interface influence nAChR function. Despite its location on the lipid-facing surface of the M4 α-helix in the α-subunit where it is distant from structures implicated in channel gating (Fig. 1)^27,28,33–35^, the C418W mutation potentiates channel function 15- to 25-fold leading to a congenital myasthenic syndrome^16^. Using a mutant cycle approach^34,36^, we show that interactions between M4 and the Cys-loop do not contribute energetically to channel function and do not facilitate C418W-induced potentiation. Instead, Trp418 interacts directly with Ser226 on the adjacent M1 α-helix leading to enhanced energetic couplings between Trp418/Thr422 on M4 and Ser226/Thr229 on M1, which in turn lead to a reorientation of M1 that stabilizes the open state. We thus identify an allosteric link that couples a structural change at the lipid-protein interface to altered channel function, in this case leading to a congenital myasthenic syndrome.

## Results

### Interactions between M4 and the Cys-loop do not facilitate C418W-induced potentiation

**Figure 2:**
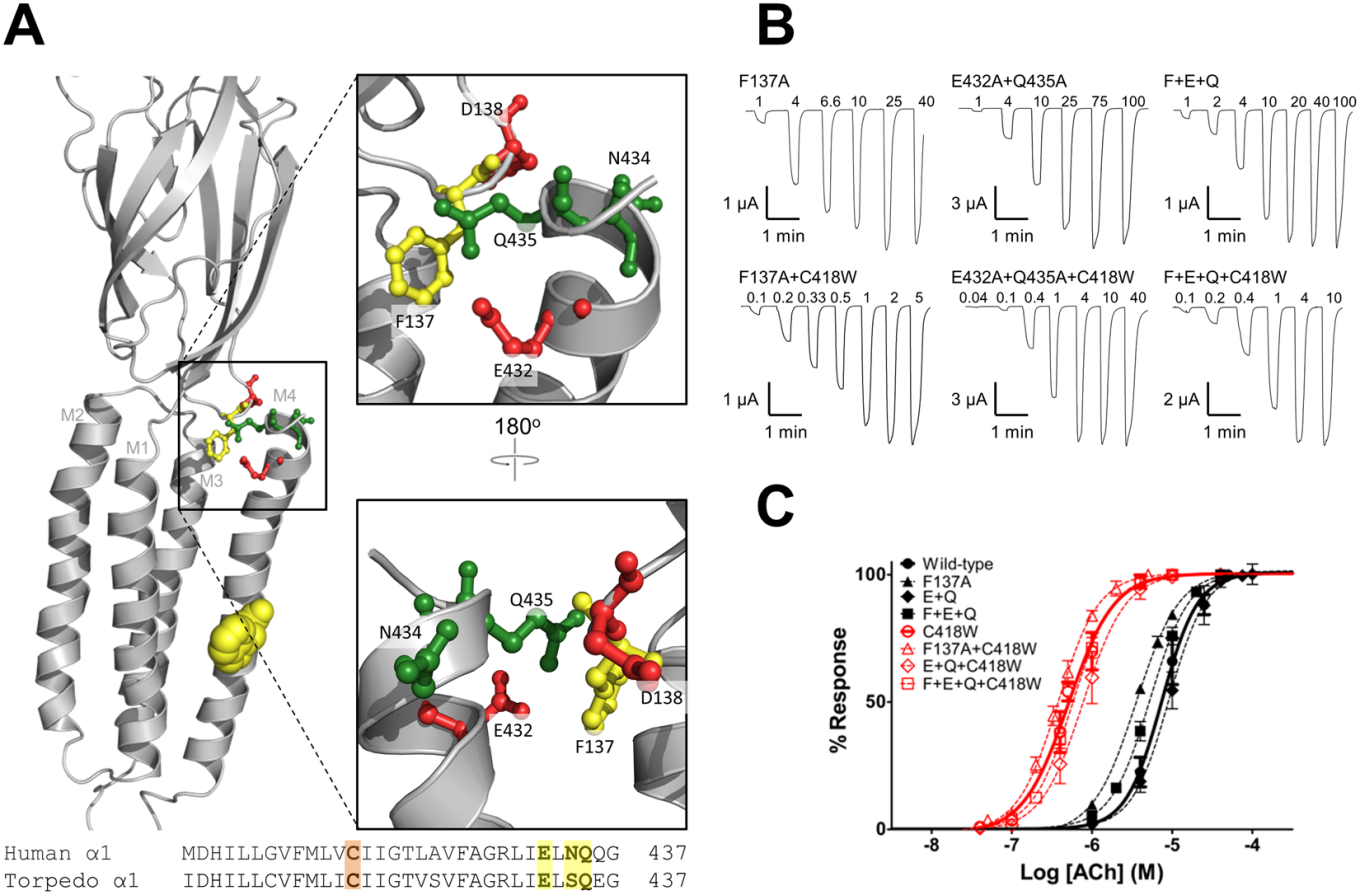
M4 - Cys-loop interactions do not play a role in C418W-induced potentiation. (**A**) Homology model of the human muscle α-subunit (based on the *Torpedo* nAChR; PDB: 2BG9), with the lipid-facing C418W mutant shown as yellow spheres. Residues involved in putative M4 - Cys-loop interactions are shown as ball and sticks where aromatic residues are yellow, polar residues are green, and negatively charged residues are red. (**B**) Representative whole cell currents elicited using the noted concentrations of acetylcholine (μM) for WT-nAChR and mutant nAChRs, where F + E + Q refers F137A + E432A + Q435A. (**C**) Averaged dose response curves showing that residues involved in putative M4 - Cys-loop interactions have minimal impact on either channel function or C418W-induced potentiation. Error bars represent standard deviation, n ≥ 8.

We first tested the hypothesis that the C418W substitution leads to a reorientation of M4 to promote interactions between M4 and the Cys-loop that enhance channel function^22^. A homology model of the human α subunit based on the *Torpedo* nAChR 4 Å resolution cryo-electron microscopic structure (PDB code: 2BG9^26^) suggests direct interactions between Glu432/Gln435 on M4 and Phe137 of the Cys-loop (Fig. 2). We also noted that a water molecule could bridge interactions between Asn434 on M4 and Asp138 on the Cys-loop. In our hands, human muscle α_2_βεδ nAChRs expressed in frog oocytes respond to acetylcholine in a dose-dependent manner characterized by an EC_50_ value of 7.61 ± 1.25 μM. Ala mutations of the three putative Cys-loop interacting residues on αM4, Q435A, N434A, and E432A had little effect on the dose response to acetylcholine, while Ala mutation of the potential M4-interacting residue on the Cys-loop, F137A, led to a slight leftward shift, corresponding to a gain-of-function (Fig. 2, Table 1). D138A did not express (Fig. S1), consistent with the important role of charged residues at the interface between the agonist-binding and TMD^37^. Even the double and triple mutants Q435A + E432A, Q435A + N434A, and Q435A + E432A + F137A had only modest effects on channel function suggesting that the observed interactions between the M4 C-terminus and the Cys-loop are not critical.

**Table 1:** Role of M4–Cys-loop interactions in nAChR function and C418W-induced potentiation.

Dose Response^a^							Potentiation (fold)^f^
Mutation(s)/Deletion(s)	WT-nAChR^c^			C418W-nAChR^d^			
	EC_50_ (μM)	Hill Slope	*n*	EC_50_ (μM)	Hill Slope	*n*	
None	7.61 ± 1.25	1.70 ± 0.47	50	0.47 ± 0.12	1.54 ± 0.23	50	16.2
F137A (Cys-loop)	3.47 ± 0.26^b^	1.44 ± 0.24	9	0.37 ± 0.04	1.67 ± 0.12	8	9.4
D138A (Cys-loop)	**No current** ^**e**^		8		1.26 ± 0.05	3	—
E432A (M4)	8.83 ± 0.62	1.92 ± 0.13	11	0.50 ± 0.12	1.45 ± 0.23	8	17.7
N434A (M4)	8.09 ± 0.68	1.94 ± 0.12	11	0.38 ± 0.11	1.57 ± 0.12	8	21.2
Q435A (M4)	8.07 ± 0.51	2.03 ± 0.12	13	0.40 ± 0.08	1.48 ± 0.09	8	20.2
E432A + Q435A	9.51 ± 1.67^b^	1.84 ± 0.31	10	0.80 ± 0.20^b^	1.63 ± 0.13	11	11.9
N434A + Q435A	7.56 ± 1.40	1.43 ± 0.14	8	0.34 ± 0.06	1.51 ± 0.21	8	22.2
F137A + E432A + Q435A	5.45 ± 0.63^b^	1.70 ± 0.09	8	0.58 ± 0.06	1.67 ± 0.15	8	9.4

^a^Measurements were performed 2 days after injection of cRNA. Error values represent standard deviation.

^b^*p* < 0.001 relative to control via one-way ANOVA followed by Dunnett’s post-hoc test.

^c^WT-nAChR contains only the mutation(s) listed under the “mutation(s)” column.

^d^C418W-nAChR contains C418W in addition to the mutation(s) listed under the “mutation(s)” column.

^e^Oocytes were tested 2–7 days after injection of 5–15 ng of mutant cRNA. D138A expression was characterized using ^125^I-bungarotoxin binding assays (Figure S1).

^f^EC_50_ of mutant on WT-nAChR background divided by EC_50_ of mutant on C418W-nAChR background.

Furthermore, the same Ala mutations had little impact on C418W-induced potentiation (Fig. 2, Table 1). The EC_50_ value for the acetylcholine-induced response decreases from 7.61 ± 1.25 μM in the WT-nAChR to 0.47 ± 0.12 μM in the C418W mutant (referred to as C418W-nAChR), which corresponds to a 16-fold potentiation of channel function. The single, double, and triple Ala mutations of potentially interacting M4 – Cys-loop residues in C418W-nAChR all had EC_50_ values similar to that of C418W-nAChR alone, with the C418W-induced potentiation of each mutant varying between ~10- and ~20-fold. Although the lack of dramatic effects suggest that M4 – Cys-loop interactions do not drive C418W-induced potentiation, interpretation of the data is complicated by the fact that changes in potentiation can be observed when calculating the ratio between the measured EC_50_ values for two mutants, even when the individual mutations yield EC_50_ values that are statistically insignificant relative to their controls, or vice versa.

To better assess the hypothesized role of M4–Cys-loop interactions in C418W-induced potentiation, the data were cast as mutant cycles. The mutant cycle allows one to determine the extent to which two or more residues energetically couple to influence channel function^34,36,38,39^. The EC_50_ values for two individual point mutations, the respective double mutation, and the wild-type are compared to calculate a value for Ω (see Experimental Methods), where an Ω value of 1 indicates that the effects of the two mutations on channel function occur independently. We considered the measured energetic couplings between two residues to be notable only when Ω differs from 1 by a factor of at least 2 (i.e. Ω ≥ 2 or Ω ≤ 1/2)^39^. In such cases, the free energy (ΔΔG) of coupling towards channel function is ≥ |1.72 kJ/mol|. A negative free energy indicates that the interaction between the residues in question is beneficial to function, while a positive free energy indicates the opposite. ΔΔG is a thermodynamic parameter that does not disclose whether the energetic coupling arises through direct or propagated interactions.

Mutant cycles suggest that neither Phe137, Glu432, Asn434, nor Gln435 are indirectly coupled with C418W (Table 2). In other words, the effect of each Ala mutation at the M4 – Cys-loop interface on channel function occurs by a mechanism that is independent of the mechanism by which C418W enhances channel function. More importantly, the two M4 residues, Q435A + E432A, are not energetically coupled with F137A of the Cys-loop in either the presence or absence of the C418W mutation, suggesting that interactions between M4 and the Cys-loop do not contribute to either channel function or C418W-induced potentiation.

**Table 2:** Energetic couplings between residues at the M4-C-terminus, the Cys-loop, and C418W.

Mutants	Ω	ΔΔG (kJ/mol)		
Phe137/Trp418	0.58	−1.35		
Asp138/Trp418	—^b^	—		
Glu432/Trp418	1.09	0.21		
Asn434/Trp418	1.31	0.67		
Gln435/Trp418	1.25	0.55		
Glu432-Gln435/Trp418	0.73	−0.78		
Asn434-Gln435/Trp418	1.37	0.78		
Phe137-Glu432-Gln435/Trp418	0.58	−1.35		
**Mutants**	**WT-nAChR** ^**c**^		**C418W-nAChR** ^**d**^	
	**Ω**	**ΔΔG (kJ/mol)**	**Ω**	**ΔΔG (kJ/mol)**
Glu432/Gln435	1.02	0.05	1.88	1.56
Asn434/Gln435	0.88	−0.32	1.05	0.12
Phe137/Glu432-Gln435	1.25	0.55	0.91	−0.23

^a^The free energy is sufficient to indicate energetic coupling.

^b^Could not calculate energetic coupling because of non-functional/non-expressing mutants.

^c^WT-nAChR contains only the mutation(s) listed under the “mutants” column.

^d^C418W-nAChR contains C418W in addition to the mutation(s) listed under the “mutation(s)” column.

Finally, given the modest resolution of the *Torpedo* nAChR structure, we tested the possibility that other residues at the M4 C-terminus interact with the Cys-loop to both influence channel function and facilitate C418W-induced potentiation by measuring the consequences of sequential M4 C-terminal deletions (Fig. S2, Table S1). Deletion of from one to four C-terminal residues (i.e. up to one full turn at the C-terminus of the M4 α-helix), including the potential Cys-loop interacting residues Gln435 (*ΔQQG*) and Asn434 (*ΔNQQG*), had little effect on the dose response with each of the four deletion mutants yielding EC_50_ values similar to that of the wild type (WT-nAChR). Subsequent deletion of the aliphatic, lipid-facing residue, Leu433, led to a slight increase in EC_50_ from 8.49 ± 1.39 μM (*ΔNQQG)* to 11.8 ± 1.0 μM (*ΔLNQQG*), representing a slight loss of channel function, while deletion of the next three residues, including Glu432, had little additional effect. Further deletions from 9 to 12 residues led to increasing right-shifts in the dose response, although even the 11-deletion mutation, *ΔAGRLIELNQQG*, exhibited only a 3-fold loss of function relative to WT-nAChR. Note that the loss-of-function phenotypes with increasing M4 C-terminal deletions correlates with a progressive loss of cell-surface expression, as measured by [^125^I]-α-bungarotoxin binding (Fig. S1). Cell-surface expression was not detectable after deletion of the final 12 C-terminal residues. The lack of a dramatic loss of function with the deletion of any particular residue suggests that none of the residues in the final three turns at the M4 C-terminus are directly involved in interactions with the remainder of the TMD that are essential to channel function. Instead, the progressive loss of function with increasing M4 C-terminal deletions may be due to a general disruption of the M4 α-helical structure/stability within the membrane.

The M4 C-terminal deletions also had minimal effects on C418W-induced potentiation (Fig. S2, Table S1). The one to four residue M4 C-terminal deletions in C418W-nAChR all had EC_50_ values similar to that of C418W-nAChR alone. Subsequent deletion of the lipid-facing Leu433 residue (*ΔLNQQG-C4*1*8W)* led to a slight reduction in C418W-induced potentiation, with further losses of potentiated function not occurring until after deletion of the final two full turns of M4. Mutant cycle analysis suggests that the functional effects of the M4 C-terminal deletions are independent of the functional effects of the C418W mutation (Table S2). The subtle changes in potentiation that occur with additional M4 C-terminal deletions could simply reflect a general destabilization of M4 structure.

In conclusion, our data show that the M4 C-terminus, and thus M4 - Cys-loop interactions, do not play a major role in either human muscle-type nAChR function or the potentiation of function that occurs with the C418W mutation.

### Channel function is sensitive to substitutions at position 418 on the M4 α-helix

An alternative hypothesis is that Trp418 interacts directly with adjacent residues leading to a change in TMD helix-helix packing that stabilizes the open state. This hypothesis implies that transmembrane helix-helix packing is sensitive to the side chain found at position 418 on the M4 α-helix. To test this hypothesis, we mutated Cys418 to a number of different residues. Mutation of Cys418 to Ala led to a slight loss-of-function (Table S3) (see also^15^). Substitution of Cys418 with the relatively large aromatic Phe, positively-charged Lys, or negatively-charged Glu residues each led to a potentiation of channel function, with the values of potentiation varying from ~3- to 5-fold relative to WT-nAChR. Although the degree of potentiation with these mutations is not as large as that seen with the αM4 C418W mutation (16-fold), the results confirm that channel function in the human muscle nAChR is sensitive to side-chain chemistry at position 418. In fact, multiple amino acid substitutions at different lipid-facing positions along M4 (Leu410, Met415, Cys418, Thr422 and Phe426) in the mouse and *Torpedo* nAChRs show that gating is more sensitive to side-chain substitutions at position 418 than at other positions along the M4 lipid-protein interface^14,40,41^.

### Interactions between Trp418 and adjacent residues influence potentiation

**Figure 3:**
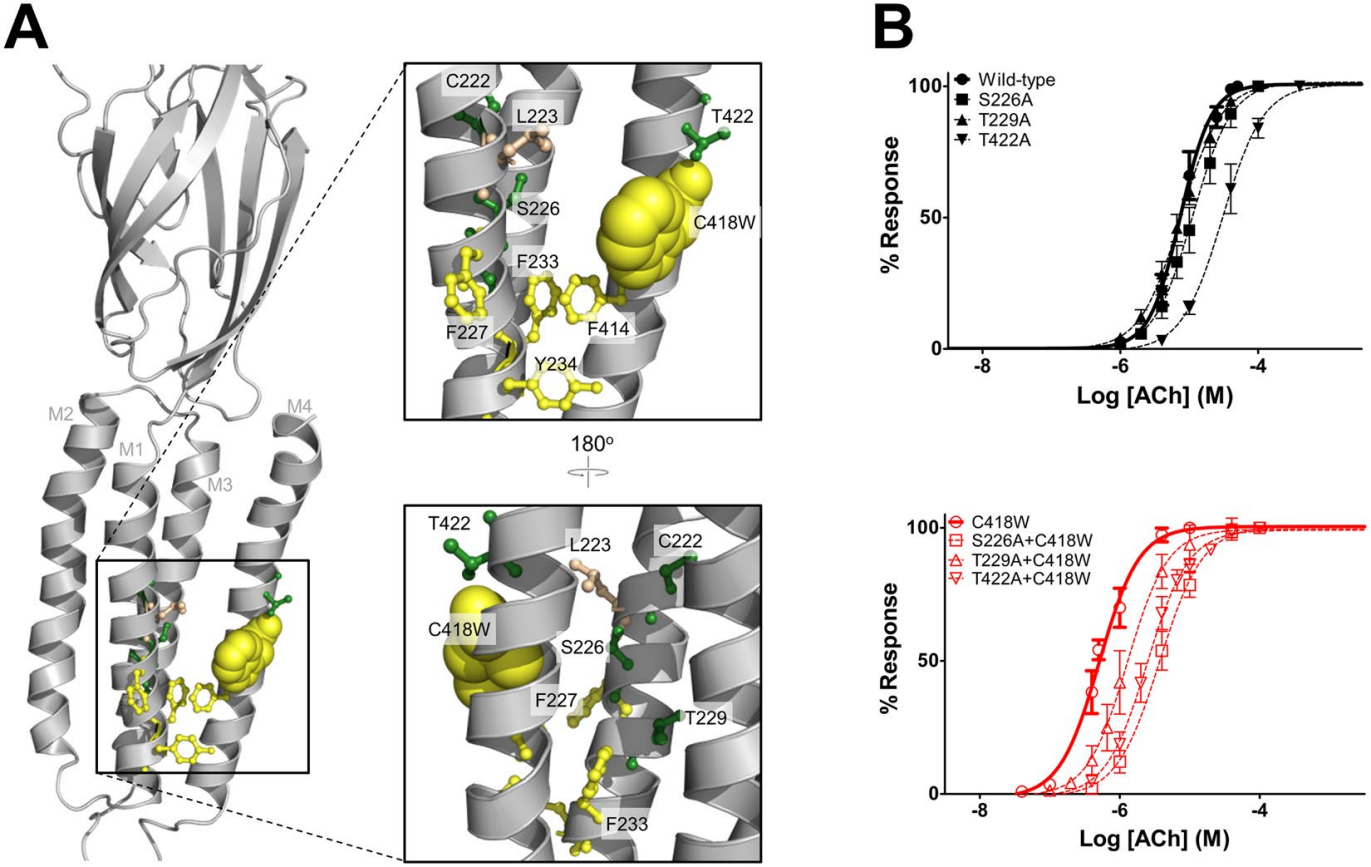
Roles of residues adjacent to C418W in potentiation. (**A**) Homology model of the human muscle-type α-subunit (based on the *Torpedo* nAChR; PDB: 2BG9), with the lipid-facing C418W mutant shown as yellow spheres. Potential interacting residues adjacent to C418W are shown as ball and sticks with aromatic residues yellow, aliphatic residues tan, and polar residues green. (**B**) Averaged dose response curves showing the effect of the individual Ala mutations of three key polar residues on the WT-nAChR background (top) and C418W-nAChR background (bottom). Error bars represent standard deviation, n ≥ 8.

Trp418 could interact directly with Leu223, Ser226 and/or Phe227 on M1, which could further facilitate interactions between the two adjacent residues on M4, Phe414 and Thr422, and Phe233/Tyr234 or Ser226 on M1, respectively, to drive C418W-induced potentiation (Fig. 3). An Ala-scan of these potentially interacting residues suggests that Ser226, Phe227 and Thr422 may be important (Table 3). The S226A mutation had little effect on the function of the WT-nAChR (less than 2-fold change in EC_50_), but led to an ~8-fold loss-of-function in the presence of Trp418, reducing C418W-induced potentiation from 16-fold down to 3-fold. F227A had no effect on channel function in the WT-nAChR, but did not express when superimposed on C418W-nAChR (i.e. F227A + C418W). If an interaction between Trp418 and Phe227 is required for folding, it seems plausible that a similar interaction could play a role in C418W-nAChR function. Finally, although T422A decreased channel function ~4-fold relative to WT-nAChR (see also^15,40^), the mutation led to a 7-fold loss-of-function in C418W-nAChR. This suggests that Thr422 becomes more important to channel activity when C418W is present. Indeed, the T422A mutation decreased C418W-induced potentiation from 16- to 9-fold.

**Table 3:** Role of residues adjacent to C418W in nAChR function and C418W-induced potentiation.

Mutation(s)	Dose Response^a^						Potentiation (fold)^f^
	WT-nAChR^c^			C418W-nAChR^d^			
	EC_50_ (μM)	Hill Slope	*n*	EC_50_ (μM)	Hill Slope	*n*	
None	7.61 ± 1.25	1.70 ± 0.47	50	0.47 ± 0.12	1.54 ± 0.23	50	16.2
C222A (M1)	4.75 ± 0.84	1.52 ± 0.15	8	0.51 ± 0.13	1.87 ± 0.15	9	9.3
L223A (M1)	**No current** ^e^		8	2.26 ± 0.43^b^	1.57 ± 0.11	10	—
S226A (M1)	12.3 ± 3.3^b^	1.52 ± 0.09	8	3.66 ± 0.82^b^	1.26 ± 0.12	8	3.4
F227A (M1)	6.51 ± 1.47	1.43 ± 0.16	8	**No current** ^e^		8	—
T229A (M1)	7.72 ± 1.37	1.40 ± 0.07	8	1.30 ± 0.33^b^	1.53 ± 0.36	8	5.9
F233A (M1)	2.63 ± 0.31^b^	1.63 ± 0.20	9	0.21 ± 0.04	1.63 ± 0.18	8	12.5
Y234A (M1)	**No current** ^e^		8	**No current** ^e^		8	—
F414A (M4)	4.47 ± 0.82	1.76 ± 0.34	10	0.32 ± 0.09	1.50 ± 0.16	8	14.0
T422A (M4)	31.2 ± 9.0^b^	1.40 ± 0.12	10	3.50 ± 1.29^b^	1.49 ± 0.14	10	8.9
S226A + T229A	11.1 ± 1.9	1.40 ± 0.08	8	7.24 ± 1.37^b^	1.48 ± 0.16	8	1.5
S226A + T422A	11.8 ± 3.3	1.41 ± 0.22	8	4.00 ± 1.39^b^	1.45 ± 0.27	7	3.0
S226A + T229A + T422A	16.6 ± 2.1^b^	1.47 ± 0.08	8	17.3 ± 3.3^b^	1.48 ± 0.11	8	1.0

^a^Measurements were performed 2 days after injection of cRNA. Error values represent standard deviation.

^b^*p* < 0.001 relative to control via one-way ANOVA followed by Dunnett’s post-hoc test.

^c^WT-nAChR contains only the mutation listed under the “mutation” column.

^d^C418W-nAChR contains C418W in addition to the mutation listed under the “mutation” column.

^e^Oocytes were tested 2–7 days after injection of 5–15 ng of mutant cRNA.

^f^EC_50_ of mutant on WT-nAChR background divided by EC_50_ of mutant on C418W-nAChR background.

In contrast, the Ala scan suggests that Phe233, Tyr234 and Phe414 are less likely to be involved (Table 3). F233A and F414A led to subtle gains-of-function when superimposed onto both WT-nAChR and C418W-nAChR, with neither leading to a substantial change in C418W-induced potentiation. Conversely, neither Y234A nor the Y234A + C418W double mutant express and/or function in frog oocytes. A Tyr residue at this position, however, is conserved in all the nAChR subunits, where it is positioned to interact with the surrounding phospholipid head groups. Ala substitutions of the same Tyr in the β-, δ-, or ε-subunits also do not express/function (data not shown), consistent with a Tyr at this position in each subunit playing a role positioning M1 within the lipid bilayer to facilitate folding.

Finally, the L223A mutation did not express/function, suggesting that the bulky aliphatic side-chain is important to folding (Table 3). Interestingly, the L223A + C418W double mutant did express, but exhibited a 5-fold loss-of-function relative to C418W-nAChR. This could indicate that Leu223 directly contributes to C418W-induced potentiation. Alternatively, the C418W mutation may rescue a functionally impaired L223A mutation. Given the data presented below, a role for Leu223 in potentiation was not pursued further.

### Phe227 is important to folding but not C418W-induced potentiation

F227A has no effect on function in WT-nAChR, but does not express when coupled with C418W, suggesting a direct link between these two aromatic residues. Given the importance of aromatic interactions at the M4-M1/M3 interface in both the folding and function of other pLGICs^23,31,42,43^, we first considered the possibility that canonical π-π or CH-π interactions between Trp418 and Phe227 lead to a local conformational change that ultimately drives C418W-induced potentiation. Substitution of Phe227 with aliphatic (Val, Leu), aromatic (Tyr, Trp), and charged (Lys, Glu) residues (Table S3) had little effect on WT-nAChR, although no ACh-induced current was observed for F227L. The same substitutions superimposed on the C418W-nAChR background had modest effects on the EC_50_ values for channel function relative to C418W-nAChR, although no current was observed for either the F227A + C418W or the F227V + C418W double mutants. Significantly, the channel potentiations observed with the Phe227“X” + C418W double mutants varied from 10- to 23-fold, relative to the 16-fold potentiation observed with C418W-nAChR. Although the lack of dramatic effects on C418W-induced potentiation suggests that π-π or CH-π interactions between Trp418 and Phe227 are not critical to C418W-induced potentiation, it is possible that these interactions are replaced by either π-π or CH-π interactions between Tyr227 or Trp227 and Trp418, cation-π interactions between Lys227 and Trp418, or anion-CH interactions between Glu227 and Trp418, consistent with the idea that attractive interactions between residues at these two locations contribute to potentiation.

To further assess whether direct interactions between Phe227 and Trp418 facilitate M1-M4 interactions to potentiate channel function, a series of double mutations was created with either like or opposite charges at these two positions (Fig. S3, Table S3). Although the F227K + C418K double mutant did not express and/or function, the F227K + C418E, F227E + C418K, and F227E + C418E double mutants led to EC_50_ values of 1.26 ± 0.36, 3.03 ± 1.10 and 2.38 ± 0.60 μM, respectively, relative to 7.61 ± 1.25 μM for the WT-nAChR, the double mutations corresponding to potentiations in channel function of 6-, 3-, and 3-fold, respectively. These data further strengthen the suggestion that local structural changes in this region influence channel function. The similar degrees of potentiation, however, particularly with the F227E + C418K and F227E + C418E double mutants, suggests that attractive interactions between residues at positions 227 and 418 are not critical to C418W-induced potentiation.

The possibility of direct interactions between Phe227 and Trp418 was explored further by casting the data as mutant cycles (Table S4). Although the energetic coupling between Phe227 and Trp418 could not be measured due to the lack of expression of the F227A + C418W double mutant, other mutant cycles suggest that there is no energetic coupling between any two residues at positions 227 and 418. Specifically, this was true with Phe/Trp located at position 227 on M1 and Phe at position 418 on M4. Even the opposite charges at positions 227 and 418 (Lys227/Glu418 and Glu227/Lys418) showed minimal if any coupling (<2.0 kJ/mol). Although an interaction between Phe227 and Trp418 in C418W-nAChR may contribute to folding, it does not drive C418W-induced potentiation.

### A cluster of polar residues at the M4-M1 interface allosterically links C418W to altered channel function

We next explored the role of polar residues adjacent to the Trp418 in C418W-induced potentiation. Both the S226A and T422A mutations reduce the degree of C418W-induced potentiation implicating both residues in the underlying mechanism (Table 3). Ser226 is energetically coupled to Trp418, contributing roughly −4 kJmol^−1^ toward channel function (Table 4). In the WT-nAChR, Ser226 and Thr422 are energetically coupled, contributing −3.64 kJmol^−1^ towards channel function. In C418W-nAChR, the coupling energy increases to −4.87 kJmol^−1^, suggesting that an interaction between these two residues is enhanced in the presence of the C418W mutation.

**Table 4:** Energetic coupling involving residues adjacent to C418W.

Mutants	Ω	ΔΔG (kJ/mol)		
Cys222/Trp418	0.58	−1.35		
Leu223/Trp418	—^b^	—		
Ser226/Trp418	0.21	−3.87^a^		
Phe227/Trp418	—^b^	—		
Thr229/Trp418	0.37	−2.46^a^		
Phe233/Trp418	0.77	−0.65		
Tyr234/Trp418	—^b^	—		
Phe414/Trp418	0.86	−0.37		
Thr422/Trp418	0.55	−1.48		
Ser226+Thr229/Trp418	0.10	−5.70^a^		
Ser226+Thr422/Trp418	0.18	−4.25^a^		
Ser226+Thr229+Thr422/Trp418	0.06	−6.97^a^		
**Mutants**	**WT-nAChR** ^c^		**C418W-nAChR** ^**d**^	
	**Ω**	**ΔΔG (kJ/mol)**	**Ω**	**ΔΔG (kJ/mol)**
Ser226/Thr229	0.89	−0.29	0.72	−0.81
Ser226/Thr422	0.23	−3.64^a^	0.14	−4.87^a^
Ser226 + Thr229/Thr422	0.36	−2.53^a^	0.32	−2.82^a^

^a^The free energy is sufficient to indicate energetic coupling.

^b^Could not calculate energetic coupling because of non-functional/non-expressing mutants.

^c^WT-nAChR contains only the mutation(s) listed under the “mutants” column.

^d^C418W-nAChR contains C418W in addition to the mutation(s) listed under the “mutation(s)” column.

**Figure 4:**
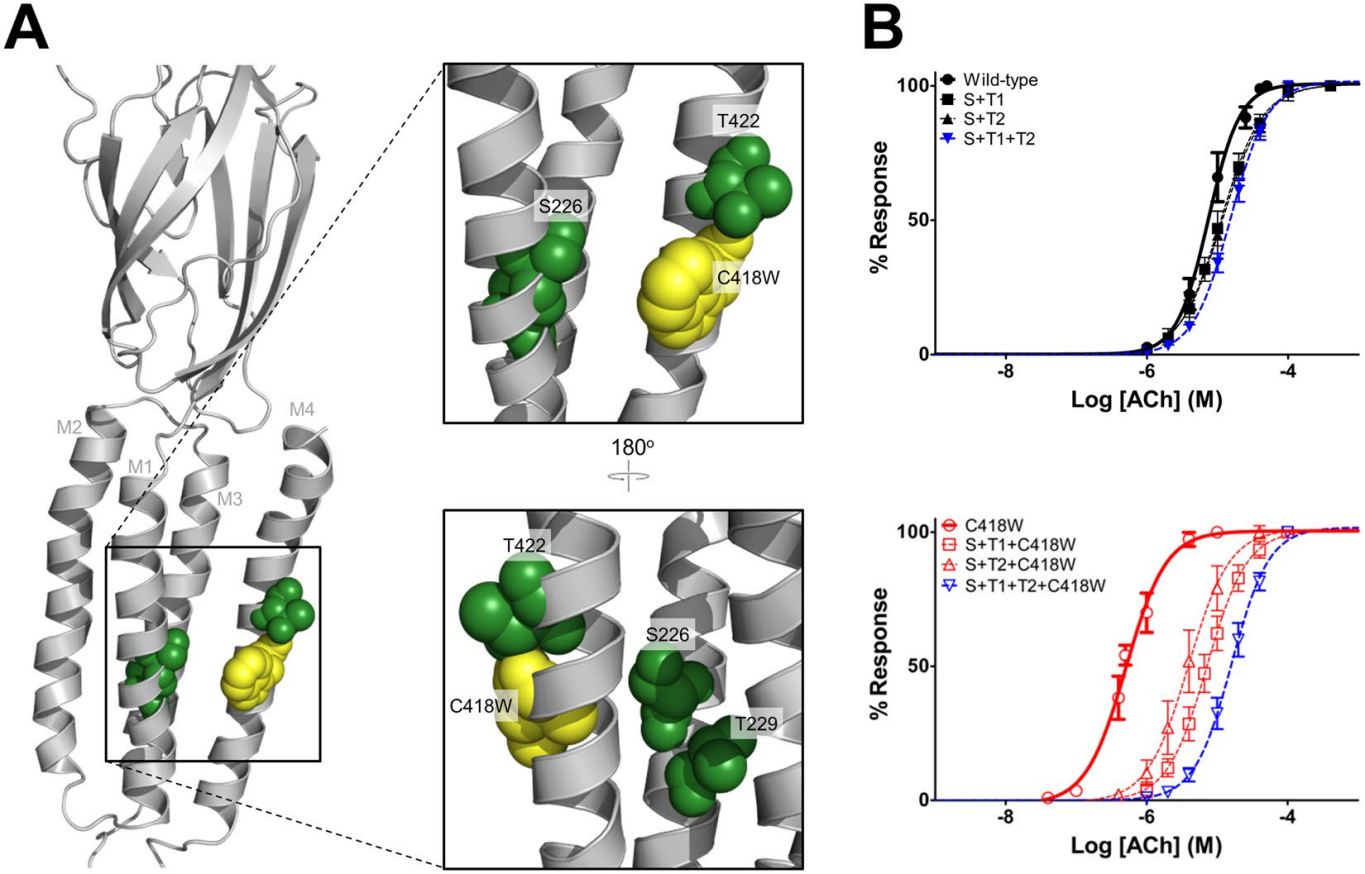
A cluster of polar residues at the M1/M4 interface is the allosteric link connecting C418W to potentiation. (**A**) Homology model of the human muscle-type α-subunit (based on the *Torpedo* nAChR; PDB: 2BG9), with the lipid-facing C418W mutant shown as yellow spheres. Three polar residues critical to potentiation are shown as green spheres. (**B**) Averaged dose response curves showing the effects of the double and triple Ala mutations of the three implicated polar residues on the WT-nAChR (top) and C418W-nAChR background (bottom). The triple Ala mutant (blue) completely abolishes C418W-induced potentiation. S226A + T229A is denoted as S + T1, S226A + T422A is denoted as S + T2, and S226A + T229A + T422A is denoted as S + T1 + T2. Error bars represent standard deviation, n ≥ 8.

We explored the possibility that residues adjacent to Ser226, Cys222 and Thr229, play a role in potentiation. The C222A and T229A mutations each led to only a slight change in the EC_50_ value in WT-nAChR. C222A had little effect on C418W-induced potentiation. In contrast, T229A reduced potentiation down to 6-fold. Thr229 couples energetically to Trp418 and contributes a modest −2.46 kJmol^−1^ toward channel function (Table 4). The double mutants S226A + T229A and S226A + T422A decrease C418W-induced potentiation from 16-fold down to 1.5- and 3.0-fold, respectively. Significantly, the S226A + T229A + T422A triple mutant completely eliminates C418W-induced potentiation (Figs 1, 4, Table 3,). In addition, interactions between these three residues and Trp418 contribute −6.97 kJmol^−1^ toward channel gating, completely accounting for C418W-induced potentiation (−6.87 kJmol^−1^). The latter shows that C418W induces potentiation of channel function by promoting local interactions between Trp418/Thr422 on M4 and Ser226/Thr229 on M1. This cluster of polar residues at the M1-M4 interface thus forms the allosteric link between the C418W mutation and altered channel function.

## Discussion

The outermost M4 α-helix in each nAChR subunit is the primary contact between the TMD and lipids, and is likely a key structural element involved in translating altered lipid-protein interactions into altered channel function. Mutations along the lipid-facing surface of M4 that alter “lipid-protein” interactions influence channel function^13–19^ despite their location peripheral to the key structures that have been implicated in channel gating^27,28,33–35^. In particular, the lipid facing C418W mutation on the αM4 α-helix of the human muscle nAChR potentiates channel function 16-fold and leads to a congenital myasthenic syndrome^16^. Here, we show for the first time how this change in structure at the lipid-nAChR interface communicates with the remainder of the TMD to ultimately alter channel function.

Although the M4 C-terminus extends beyond the lipid bilayer to interact directly with the Cys-loop, a key structure implicated in channel gating, our data show that M4 – Cys-loop interactions are not critical to function and do not facilitate C418W-induced potentiation. Ala mutations of potential interacting residues at the interface between M4 and the Cys-loop have little impact. There is no energetic coupling between these residues that contributes to channel function, whether or not the C418W mutation is present. M4 C-terminal deletions, which would eliminate any potential M4 - Cys-loop interacting residues, have minimal effects on either function or C418W-induced potentiation. Furthermore, even the subtle functional consequences of these C-terminal deletions occur through a mechanism that is independent of the mechanism by which C418W leads to enhanced channel function.

These data show that the M4 C-terminus of the nAChR is not implicated in channel function, in stark contrast to what is observed with other pLGICs. In GLIC, mutations or deletions involving the final four residues at the M4 C-terminus dramatically inhibit channel activity, presumably due to the elimination of contacts between M4 and both the adjacent M3 α-helix and the Cys-loop equivalent, the β6-β7 loop^32^. Similarly, deletion of the final four C-terminal residues in the human GABAρ1 receptor causes a profound 1000-fold loss-of-function, with further deletion of the fifth residue resulting in the loss of channel activity^44^.

On the other hand, analogous functionally important interactions between M4 and the β6-β7 loop are not found in ELIC^32^. In fact, pLGICs exhibit two distinct TMD archetypes, one where there are tight interactions between M4 and both M1/M3 and the Cys-loop driven by an extensive network of aromatic residues that is essential for channel function, and another archetype where such interactions are less important^43^. Sequence comparisons suggest that the nAChR belongs to the second archetype, where interactions between M4 and both M1/M3 and the Cys-loop are not essential, consistent with the findings presented here.

So how does C418W potentiate channel function? By Ala scanning mutagenesis and mutant cycle analyses, we show that the Trp at position 418 interacts energetically with an adjacent Ser residue at the M1 – M4 interface, and that these interactions facilitate additional energetic couplings involving Thr422 on M4 and Thr229 on M1 to facilitate channel function. This conclusion is based on the following observations: First, channel function is sensitive to side chain substitutions at these and adjacent positions on at the M1 - M4 interface, indicating that human muscle nAChR function is sensitive to altered helix-helix packing in this region. Second, individual Ala mutations of Ser226, Thr229 and Thr422 all diminish the extent of C418W-induced potentiation. Third, Ser226, Thr229 and Thr422 couple energetically with Trp418, with the energetic couplings contributing −3.87, −2.46 and −1.48 kJmol^−1^ to enhance channel function. In fact, interactions between these three polar residues and Trp418 collectively contribute −6.97 kJmol^−1^ towards enhanced channel function, thus fully accounting for C418W-induced potentiation (−6.87 kJmol^−1^). Finally, the triple Ala mutant, S226A + T229A + T422A, completely abolishes C418W-induced potentiation. C418W-induced potentiation is thus initiated by enhanced energetic couplings between Trp418/Thr422 on M4 and Ser226/Thr229 on M1.

As polar residues are key drivers of helix-helix interactions in the hydrophobic membrane environment^45^, it seems likely that the enhanced interactions between Trp418/Thr422 on M4 and Ser226/Thr229 on M1 lead to/result from a reorientation of both the M1 and M4 α-helices. Given that the C-terminus of M4 is not implicated, potentiation is likely manifest entirely by a reorientation that alters interactions between M1 and structures implicated in channel gating. A reorientation of M1 could alter direct interactions between M1 and the pore-lining M2 α-helix to stabilize M2 in the open state. Alternatively or in addition, a reorientation of M1 could lead to a subtle change in conformation of pre-M1, a structure important to channel gating^28,34^. A change in conformation of pre-M1 could influence interactions between a plethora of residues at the interface between the extracellular agonist-binding and the TMD to influence channel function. A change in orientation of M1 could lead to altered energetic couplings between a vast number of residues in the TMD to stabilize the open state.

Many positive allosteric modulators, including neurosteroids, anaesthetics, and alcohols bind to inter- and intra-subunit cavities within the TMD of various pLGICs to modulate their activity^46–51^. Binding sites are typically located near the extracellular end of the TMD where the binding molecules can interact directly with the Cys-loop or other residues implicated in channel function. As demonstrated by the α7 nAChR-specific potentiator, PNU-120596, however, modulation of activity can occur through interactions with other residues not directly implicated in channel function^49^. In these cases, the drugs presumably alter gating by generally perturbing the TMD structure, as appears to be the case with the C418W mutation. C418W may potentiate muscle-type nAChR activity in a manner similar to PNU-120596 in that the mutation modifies central contacts between TMD α-helices. It has been suggested that the intra-subunit cavity occupied by PNU-120596 may be a highly conserved modulatory site in Cys-loop receptors^47^.

Finally, although the M4 C-terminal deletions do not reveal a critical functional role for the M4 C-terminus, they do lead to a progressive loss of cell surface expression. Deletion of the final 12 residues at the M4 C-terminus, and thus three complete α-helical turns, abolishes expression of the nAChR as measured by [^125^I]-α-bungarotoxin binding. The 12^th^ residues, Phe426, is conserved amongst all muscle-type subunits and protrudes toward the lipids^26,52^, where it may interact with the polar phospholipid head groups^53^. It is possible that this residue is critical for positioning M4 within the lipid bilayer parallel to M1/M3. The elimination of Phe426 may hinder folding of M4, which could unmask an endoplasmic reticulum retention motif located N-terminal to the adjacent M1 helix thus resulting in internal degradation^54^. Our findings are consistent with several other studies, which have highlighted a role for M4 in folding, assembly, and trafficking. Shortening M4 in the *Torpedo* nAChR reduces expression in frog oocytes^29^, with positively-charged residues flanking M4 playing an important role^55^. A truncation of the εM4 C-terminus also abrogates cell surface expression leading to a congenital myasthenic syndrome^56^. It is thus clear that the C-terminus of αM4 is critical for cell surface expression.

In conclusion, although the functional sensitivity of the nAChR to lipids has been studied for over 30 years, little insight has been obtained into how changes at the lipid-protein interface are translated through the TMD to ultimately influence nAChR function. Here, we elucidate an allosteric link between the lipid-protein interface and altered nAChR function. We show that a disease-causing mutation along the lipid-facing surface of the M4 α-helix leads to a change in orientation of the outer most M4 α-helix that enhances interactions with the adjacent M1 α-helix. These enhanced interactions likely lead to a reorientation of M1 to stabilize the open state. Further application of this mutant cycle approach will allow us to completely map the allosteric pathway leading from the C418W mutation to altered channel function and thus ultimately to a congenital myasthenic syndrome.

## Materials and Methods

### cRNA constructs for oocyte expression

Wild-type human α_1_, β_1_, δ, and ε nAChR-pRBG4 clones were kindly provided by Steven Sine. Each nAChR subunit DNA was transferred into the pcDNA3 vector as an EcoRI fragment. The α_1_, β_1_, δ, and ε nAChR-pcDNA3 was linearized with XhoI and capped cRNA was produced by *in vitro* transcription using the mMESSAGE mMACHINE® T7 kit (Ambion). All mutants were created using QuikChange™ Site-Directed Mutagenesis kits (Agilent) and verified by sequencing.

### Electrophysiology

Stage V-VI oocytes^57^ were injected with 5 ng of mutated α_1_ subunit cRNA along with 2.5 ng each of wild-type β_1_, δ, and ε subunit cRNA. After incubation for ~2 days, the injected oocytes were placed in the RC-1Z oocyte chamber (Harvard Apparatus; Hamden, CT) containing HEPES buffer (96 mM NaCl, 2 mM KCl, 1.8 mM BaCl_2_, 1 mM MgCl_2_, 10 mM HEPES, pH 7.3). Whole cell currents were recorded using a two-electrode voltage clamp (TEVC) apparatus (OC-725C oocyte clamp; Holliston, MA) in the presence of 1 μM atropine to prevent activation of endogenous calcium-activated chloride channels via muscarinic acetylcholine receptors. Currents through the membrane in response to increasing acetylcholine concentrations were measured with the transmembrane voltage clamped at −60 mV for the mutants which did not contain the potentiating αM4 C418W mutation and -30 mV for the mutants which did contain the potentiating αM4 C418W mutation. Dose responses for each mutant were acquired from at least two different batches of oocytes and were repeated at least 8 times wherever possible. Each individual dose-response was fitted with a variable slope sigmoidal dose-response using GraphPad Prism and the individual EC_50_ values and Hill coefficients from each experiment averaged to give the presented values ± standard deviation.

### [^125^I]-α-bungarotoxin binding

Binding experiments were performed on intact oocytes 3 and 4 days after injection with 50 ng of mutated α_1_ subunit cRNA and 25 ng each of the wild-type non-α_1_ subunits. Up to 8 oocytes were incubated in 600 μL of 2.5 nM of [^125^I]-α-bungarotoxin and 1 mg/mL of BSA in MOR2 buffer (82 mM NaCl, 2.5 mM KCl, 5 mM MgCl_2_, 1 mM NaH_2_PO_4_, 5 mM HEPES, and 0.2 mM CaCl_2_, pH 7.4) for 2 hours at room temperature. Binding was terminated by washing the oocytes 4 times with MOR2 buffer and then quantified by γ counting. Non-specific binding was determined by incubating mock injected oocytes under identical conditions.

### Mutant Cycle Analysis

The EC_50_ values obtained from the mutagenesis experiments were cast as mutant cycles:

$${\Omega }=\,\frac{(E{C}_{50}(mu{t}_{1,2}))\ast (E{C}_{50}(WT))}{(E{C}_{50}(mu{t}_{1}))\ast (E{C}_{50}(mu{t}_{2}))}$$

where *WT* is the wild type control, *mut*_*1*_ is the first mutant, *mut*_2_ is the second mutant, and *mut*_*1,2*_ is the double mutant. The free energy of coupling (ΔΔG) was defined as:

$${\Delta }{\Delta }G=RTln({\Omega })$$

where *R* is the gas constant (8.314 J/mol*K) and *T* is the temperature constant (298 K). The mutations perform here are distant from the ACh binding sites, thus changes in EC_50_ values likely reflect changes to the gating equilibrium constant.

## Electronic supplementary material

Supplementary Information
