# Supplementary material for: An allosteric link connecting the lipid-protein interface to the gating of the nicotinic acetylcholine receptor
Source: Sci Rep. 2018 Mar 1;8:3898. doi: 10.1038/s41598-018-22150-x (PMC5832824; doi:10.1038/s41598-018-22150-x)
Supplement: Supplementary file 1 — Supplementary Information [file 41598_2018_22150_MOESM1_ESM.pdf]

Supplemental Information

An allosteric link connecting the lipid-protein interface to the gating of the nicotinic acetylcholine receptor

Jaimee A. Domville and John E. Baenziger*

Department of Biochemistry, Microbiology, and Immunology, 451 Smyth Rd., Ottawa, ON, K1H 8M5

Correspondence should be addressed to John Baenziger at the above address. E-mail John.Baenziger@uottawa.ca; Telephone: (613) 562-5800 x8222

Includes: Supplementary Data Tables S1-S4 and Figures S1-S3

Table S1. Role of M4–Cys-loop interactions in nAChR function and C418W-induced potentiation.[a]

Mutation(s)/Deletion(s)	Dose Response[b]						Potentiation (fold)[g]
	WT-nAChR[d]			C418W-nAChR[e]			
	EC$_{50}$ (μM)	Hill Slope	*n*	EC$_{50}$ (μM)	Hill Slope	*n*	
None	7.61 ± 1.25	1.70 ± 0.47	50	0.47 ± 0.12	1.54 ± 0.23	50	16.2
ΔG	6.86 ± 0.86	2.66 ± 0.67	9	0.49 ± 0.15	1.66 ± 0.14	10	14.0
ΔQG	6.37 ± 0.94	2.62 ± 0.54	9	0.50 ± 0.18	1.36 ± 0.30	8	12.7
ΔQQG	7.14 ± 1.09	2.36 ± 0.38	8	0.69 ± 0.23	1.38 ± 0.23	8	10.3
ΔNQQG	8.49 ± 1.39	2.13 ± 0.44	8	0.76 ± 0.21	1.36 ± 0.17	9	11.2
ΔLNQQG	11.8 ± 1.0[c]	1.65 ± 0.33	10	1.29 ± 0.37[c]	1.55 ± 0.21	9	9.1
ΔELNQQG	12.3 ± 1.2[c]	1.59 ± 0.25	10	1.28 ± 0.13[c]	1.62 ± 0.18	9	9.6
ΔIELNQQG	12.7 ± 1.7[c]	1.54 ± 0.15	10	1.47 ± 0.12[c]	1.71 ± 0.37	9	8.6
ΔLIELNQQG	14.7 ± 2.6[c]	1.46 ± 0.29	10	1.42 ± 0.25[c]	1.70 ± 0.08	9	10.4
ΔRLIELNQQG	14.9 ± 2.5[c]	1.77 ± 0.35	10	1.65 ± 0.34[c]	1.63 ± 0.21	9	9.0
ΔGRLIELNQQG	21.4 ± 4.2[c]	1.35 ± 0.12	10	1.87 ± 0.11[c]	1.76 ± 0.24	9	11.4
ΔAGRLIELNQQG	23.0 ± 5.0[c]	1.69 ± 0.36	10	2.88 ± 0.54[c]	1.72 ± 0.25	9	8.0
ΔFAGRLIELNQQG	**No current**[f]		10	3.92 ± 0.35[c]	1.39 ± 0.12	3	--

[a] Table S1 relates to Table 1, Figure S1, and Figure S2.
[b] Measurements were performed 2 days after injection of cRNA. Error values represent standard deviation.
[c] $p < 0.001$ relative to control via one-way ANOVA followed by Dunnett's post-hoc test.
[d] WT-nAChR contains only the mutation(s) listed under the "mutation(s)" column.
[e] C418W-nAChR contains C418W in addition to the mutation(s) listed under the "mutation(s)" column.
[f] Oocytes were tested 2-7 days after injection of 5-15 ng of mutant cRNA.
[g] EC$_{50}$ of mutant on WT-nAChR background divided by EC$_{50}$ of mutant on C418W-nAChR background.

Table S2. Energetic coupling between M4 C-terminal deletions and C418W.[a]

**Mutants**	**Ω**	**ΔΔG (kJ/mol)**
ΔG/Trp418	0.86	-0.37
ΔQG/ Trp418	0.79	-0.58
ΔQQG/ Trp418	0.64	-1.11
ΔNQQG/ Trp418	0.69	-0.92
ΔLNQQG/ Trp418	0.56	-1.44
ΔELNQQG/ Trp418	0.59	-1.31
ΔIELNQQG/ Trp418	0.53	-1.57
ΔLIELNQQG/ Trp418	0.64	-1.11
ΔRLIELNQQG/ Trp418	0.56	-1.44
ΔGRLIELNQQG/ Trp418	0.71	-0.85
ΔAGRLIELNQQG/ Trp418	0.49	-1.77[b]
ΔFAGRLIELNQQG/ Trp418	**--**[c]	**--**

[a] Table S2 relates to Table 2.
[b] The free energy is sufficient to indicate energetic coupling.
[c] Could not calculate energetic coupling because of non-functional/non-expressing mutants.

Table S3. Role of interactions between residues at position 227 and 418 in nAChR function and C418W-induced potentiation.[a]

	Dose Response[b]							
Mutation(s)	WT-nAChR[d]			Potentiat. (fold)	C418W-nAChR[e]			Potentiat. (fold)
	EC_{50} (μM)	Hill Slope	*n*		EC_{50} (μM)	Hill Slope	*n*	
None	7.61 ± 1.25	1.70 ± 0.47	50	--	0.47 ± 0.12	1.54 ± 0.23	50	16.2
C418A	10.6 ± 2.9[c]	1.82 ± 0.33	9	0.7[h]	--	--	--	--
C418F	1.67 ± 0.66[c]	1.58 ± 0.25	8	4.6[h]	--	--	--	--
C418K	3.06 ± 0.62[c]	1.27 ± 0.06	8	2.5[h]	--	--	--	--
C418E	1.66 ± 0.65[c]	1.58 ± 0.25	8	4.6[h]	--	--	--	--
F227A	6.51 ± 1.47	1.43 ± 0.16	8	--	**No current[f]**		8	--
F227V	14.8 ± 3.8[c]	1.42 ± 0.11	8	--	**No current[f]**		8	--
F227L	**No current[f]**		8		0.92 ± 0.18[c]	1.46 ± 0.20	8	--
F227Y	5.21 ± 0.80[c]	1.44 ± 0.11	8	--	0.47 ± 0.08	1.61 ± 0.15	8	11.1[g]
F227W	5.77 ± 0.71	1.45 ± 0.16	8	--	0.25 ± 0.03[c]	1.66 ± 0.08	8	23.1[g]
F227K	8.82 ± 1.13	1.37 ± 0.13	9	--	0.76 ± 0.15[c]	1.58 ± 0.06	8	11.6[g]
F227E	7.85 ± 1.97	1.54 ± 0.21	8	--	0.46 ± 0.10	1.56 ± 0.07	8	17.1[g]
F227A+C418A	12.2 ± 1.8[c]	1.38 ± 0.12	8	1.6[h]	--	--	--	--
F227A+ C418F	1.48 ± 0.35[c]	1.49 ± 0.11	8	5.1[h]	--	--	--	--
F227A+ C418K	1.73 ± 0.47[c]	1.47 ± 0.05	8	4.4[h]	--	--	--	--
F227A+ C418E	2.01 ± 0.54[c]	1.53 ± 0.19	8	3.8[h]	--	--	--	--
F227A+C418W	**No current[f]**		8	--	--	--	--	--
F227K+ C418K	**No current[f]**		8	--	--	--	--	--
F227E+ C418E	2.38 ± 0.60[c]	1.46 ± 0.11	8	3.2[h]	--	--	--	--
F227K+ C418E	1.26 ± 0.36[c]	1.52 ± 0.27	8	6.0[h]	--	--	--	--
F227E+ C418K	3.03 ± 1.10[c]	1.41 ± 0.19	8	2.5[h]	--	--	--	--
F227W+ C418F	1.15 ± 0.33[c]	1.59 ± 0.13	8	6.6[h]	--	--	--	--

[a] Table S3 relates to Table 3 and Figures S3.
[b] Measurements were performed 2 days after injection of cRNA. Error values represent standard deviation.
[c] $p < 0.001$ relative to control via one-way ANOVA followed by Dunnett's post-hoc test.
[d] WT-nAChR contains only the mutation listed under the "mutation" column.
[e] C418W-nAChR contains C418W in addition to the mutation listed under the "mutation" column.
[f] Oocytes were tested 2-7 days after injection of 5-15 ng of mutant cRNA.
[g] EC_{50} of mutant on WT-nAChR background divided by EC_{50} of mutant on C418W-nAChR background.
[h] Wild-type EC_{50} divided by mutant EC_{50}.

Table S4. Energetic coupling involving residues at positions 227 and 418.[a]

Mutants	Ω	ΔΔG (kJ/mol)
Phe227/Cys418	1.35	0.74
Phe227/Phe418	0.97	-0.08
Phe227/Lys418	1.51	1.02
Phe227/Glu418	0.71	-0.85
Phe227/Trp418	--[c]	--
Lys227/Lys418	--[c]	--
Glu227/Glu418	0.52	-1.62
Lys227/Glu418	0.46	-1.92[b]
Glu227/Lys418	0.55	-1.48
Trp227/Phe418	0.88	-0.32
Phe227/Trp418	--[c]	--

[a] Table S4 relates to Table 4.
[b] The free energy is sufficient to indicate energetic coupling.
[c] Could not calculate energetic coupling because of non-functional/non-expressing mutants.

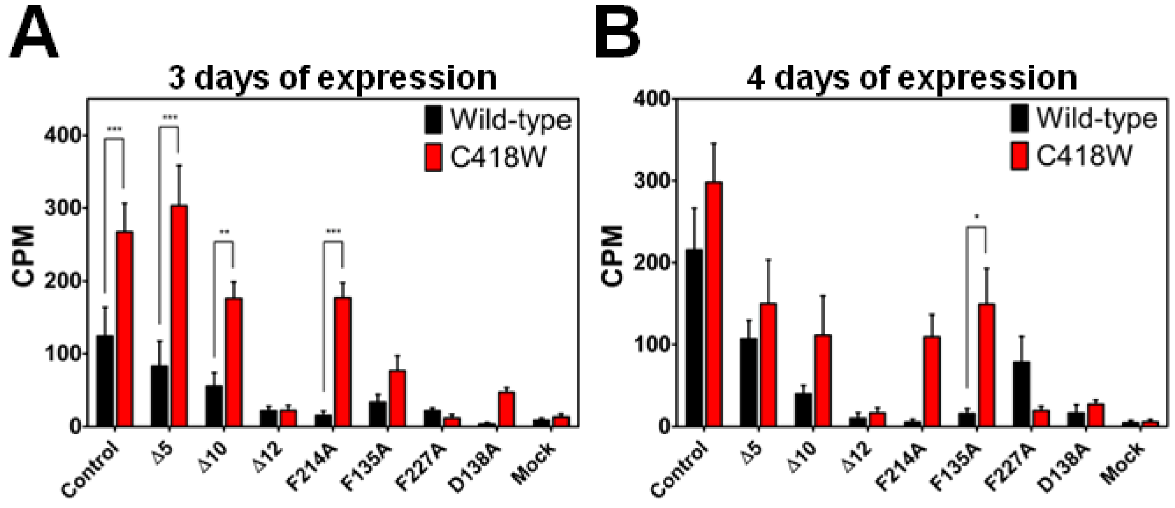

Figure S1 - Surface expression levels of select mutants as measured by [^{125}I]-α-bungarotoxin binding. Cell surface binding of [I^{125}]-α-bungarotoxin as detected by γ counting three days **A)** and four days **B)** after injection of 50 ng of either wild-type or mutant cRNA. Δ5, Δ10, and Δ12 represent deletion of 5, 10, and 12 M4 C-terminal residues, respectively. Mock oocytes were injected with buffer that did not contain any cRNA, n ≥ 8. Figure S1 relates to Figure 2, Table S1, and Figure S2.

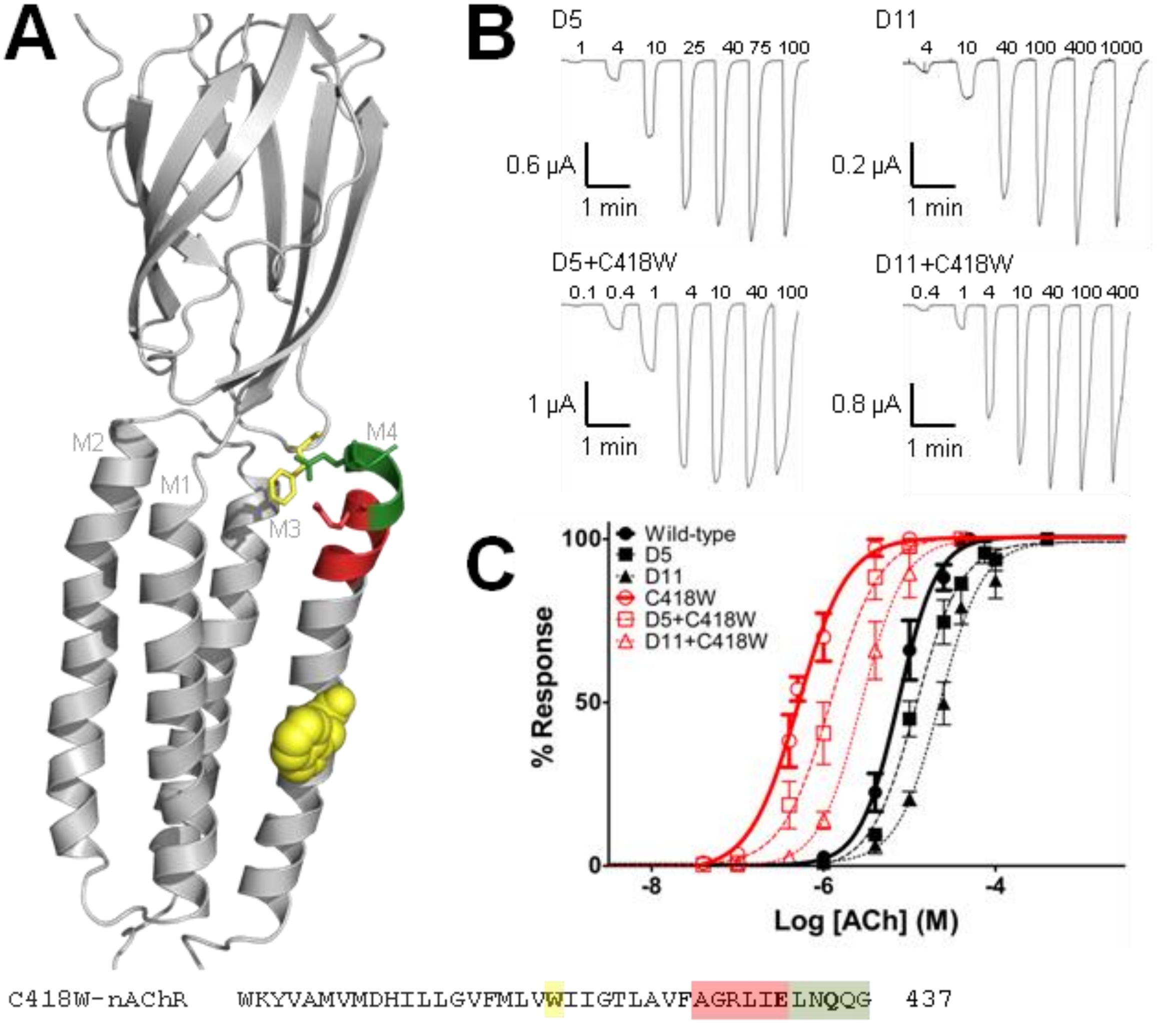

Figure S2. M4 C-terminal deletions show that the M4 C-terminus does not play a role in C418W-induced potentiation.
A) Homology model of the human muscle-type α-subunit (based on the *Torpedo* nAChR; PDB: 2BG9). The lipid-facing C418W mutant is shown as yellow spheres, Phe137 is shown as yellow sticks, the five C-terminal residues are coloured green with Gln435 shown as sticks, and the next six residues are coloured red with Glu432 shown as sticks. **B)** Two-electrode data for the deletion of 5 C-terminal residues (D5) and 11 C-terminal residues (D11) on the WT-nAChR (top) and the C418W-nAChR (bottom) backgrounds. **C)** Averaged dose response curves for D5 and D11 on the WT-nAChR versus C418W-nAChR backgrounds. Error bars represent standard deviation, n ≥ 8. Figure S2 relates to Figure 2, Table S1, and Figure S1.

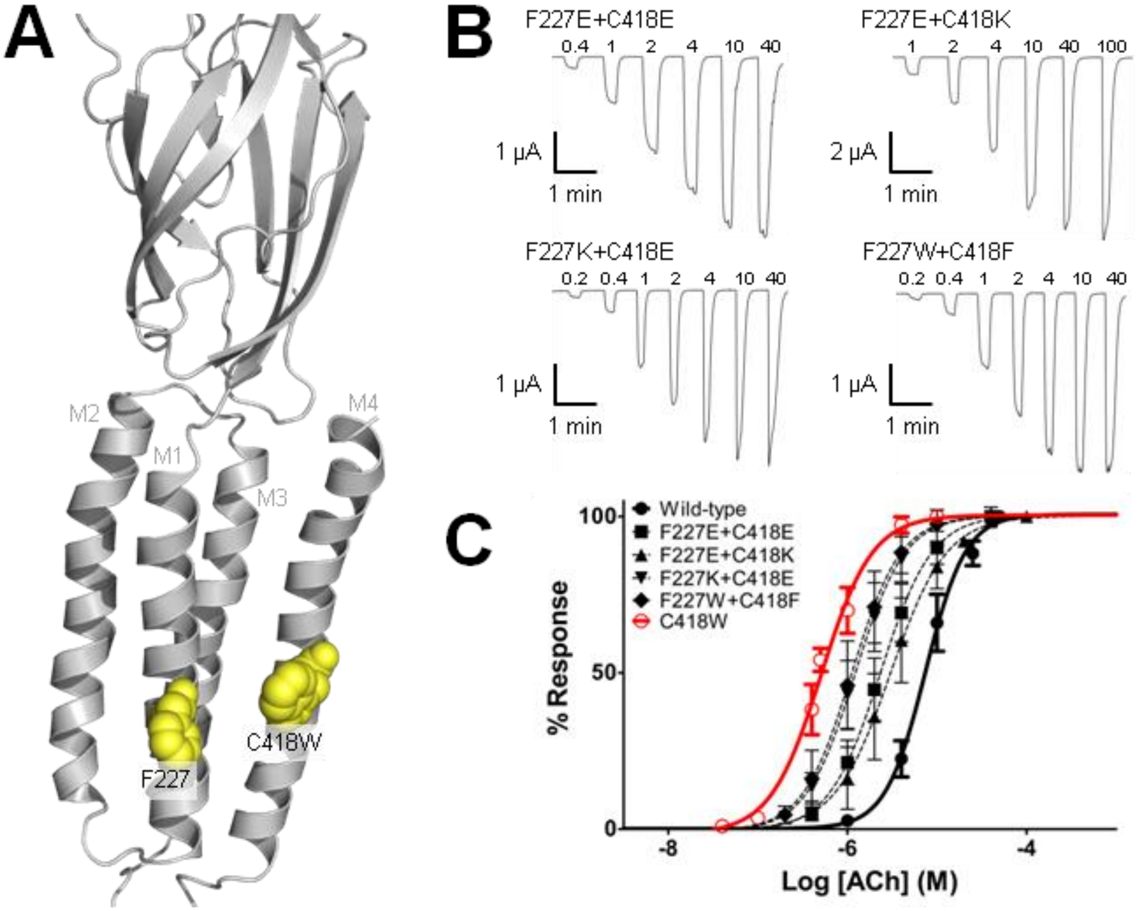

Figure S3. Interactions between residues at position 227 and 418 do not drive potentiation. **A)** Homology model of the human muscle-type α-subunit (based on the *Torpedo* nAChR; PDB: 2BG9). The lipid-facing C418W mutant and Phe227 are shown as yellow spheres. **B)** Two-electrode data for select double mutants at positions 227 and 418. **C)** Averaged dose response curves showing that select double mutants at positions 227 and 418 have an EC_{50} that falls within those of the WT-nAChR and C418W-nAChR. Error bars represent standard deviation, $n \geq 8$. Figure S3 relates to Figure 3, Table 3, and Table S3.
